# Food Additive Mixtures, Glucose Metabolism, and Type 2 Diabetes Mellitus Risk: Mechanistic Insights from Epidemiology, Human Intervention Studies, and Experimental Models

**DOI:** 10.3390/nu18152521

**Published:** 2026-08-04

**Authors:** Roko Santic, Marko Kumric, Nikola Pavlovic, Josko Bozic

**Affiliations:** 1 Department of Pathophysiology, University of Split School of Medicine, Soltanska 2A, 21000 Split, Croatia; roko.santic@mefst.hr (R.S.); marko.kumric@mefst.hr (M.K.); nikola.pavlovic@mefst.hr (N.P.); 2 Laboratory for Cardiometabolic Research, University of Split School of Medicine, Soltanska 2A, 21000 Split, Croatia

**Keywords:** type 2 diabetes mellitus, insulin resistance, food additive mixtures, dietary emulsifiers, non-nutritive sweeteners, glucose metabolism, ultra-processed foods, gut microbiota, intestinal permeability, metabolic endotoxaemia

## Abstract

Food additives are consumed as mixtures in ultra-processed foods, but their independent contribution to glucose dysregulation is difficult to separate from diet quality and food matrix. PubMed/MEDLINE, Embase, Web of Science Core Collection, Scopus, and CENTRAL were searched from inception to 30 April 2026, followed by a supplementary PubMed update on 26 July 2026. This critical narrative review integrates epidemiological, human-intervention, animal, ex vivo, and mechanistic evidence. Successive NutriNet-Santé analyses associate several additive co-exposure profiles, emulsifiers, preservatives, food colouring additives, and non-nutritive sweeteners with incident type 2 diabetes mellitus. However, these analyses use substantially overlapping participants, lack independent additive-specific cohort replication, and remain vulnerable to residual confounding. Experimental evidence is strongest for selected emulsifiers and sweeteners, which may alter gut microbiota, intestinal barrier function, inflammatory signalling, or short-term glycaemic responses. No human trial has demonstrated the complete pathway from additive exposure to clinically meaningful insulin resistance or diabetes, and null or compound-specific findings argue against a uniform class effect. Current evidence therefore supports biological plausibility, not causality or additive-specific clinical recommendations. Longer controlled feeding trials should test realistic mixtures while holding the food matrix constant and should incorporate exposure validation, repeated microbiome and metabolomic sampling, intestinal permeability measures, and validated insulin-sensitivity endpoints.

## 1. Introduction

Approximately 589 million adults aged 20–79 years live with diabetes worldwide as of the 2024 IDF estimate, with projections rising to 853 million by 2050 [1]; type 2 diabetes mellitus (T2D) accounts for the majority of these cases. While excess energy intake, refined carbohydrate load, and physical inactivity remain the dominant modifiable risk factors, they do not fully account for the accelerating prevalence observed across socioeconomically diverse populations over recent decades [2]. An underexplored dietary exposure dimension concerns food additives—substances intentionally incorporated into processed foods during manufacture for purposes of texturisation, emulsification, preservation, acidification, or sweetening—that are consumed daily at dose levels and in combinations that have no precedent in traditional diets.

Ultra-processed foods (UPFs), defined within NOVA—a food-processing classification system based on the nature, extent, and purpose of processing—as industrially formulated products characterised by minimal whole-food ingredients and a distinctive additive profile [3], are now linked to T2D by consistent epidemiological evidence. A 2024 umbrella review of 45 meta-analyses identified Class I evidence for a dose–response relationship between UPF consumption and T2D incidence, with a relative risk of 1.12 per 10% increment in dietary UPF share [4]. Three large US prospective cohorts—the Nurses’ Health Study, Nurses’ Health Study II, and the Health Professionals Follow-up Study—reported a hazard ratio (HR) of 1.46 (95% CI 1.39–1.54) comparing the highest with the lowest UPF quintile [5]. A parallel systematic meta-analysis reached concordant conclusions [6]. Yet a persistent methodological obstacle has limited progress: UPF classification conflates at least four biologically distinct exposure types—excess energy density, fibre and micronutrient displacement, processing-derived contaminants, and the additive load itself. Separating the additive contribution from these concurrent attributes has, until recently, been methodologically intractable.

UPFs and additive mixtures are related but non-equivalent exposure concepts. NOVA classifies foods according to the nature and extent of industrial processing, whereas mixture analysis estimates correlated intakes of named additives across foods. A mixture score may therefore identify co-exposure patterns within or across product categories, but it does not remove the energy density, nutrient profile, physical structure, or other matrix characteristics of the foods that deliver those additives.

Recent broader reviews of the UPF–microbiome relationship have addressed ultra-processed foods generically, treating the whole food category as the exposure unit [7]. This review distinguishes itself in three ways: it takes additive mixtures, rather than either single compounds or whole UPFs, as the primary exposure unit; it uses the broader IRS-1/IRS-2 serine/threonine phosphorylation network as a mechanistic framework through which inflammatory and nutrient-excess signals may modify insulin action, while treating Ser307/Ser312 as one context-dependent node rather than a universally inhibitory switch; and it treats host susceptibility as an explicit effect modifier rather than a nuisance variable. An important advance came in 2025 with the application of nonnegative matrix factorisation (NMF) to detailed dietary recall data from 108,643 NutriNet-Santé participants [8]. Rather than examining additives one at a time, this approach identifies latent co-occurrence patterns—real-world mixtures of additives consumed together through habitual dietary choices. Two of five identified mixture profiles remained statistically associated with T2D incidence after adjustment for measured dietary quality, total energy intake, and NOVA classification [8]. This robustness to adjustment does not demonstrate independence from unmeasured dietary-pattern or food-matrix effects. Accordingly, the mixture-based approach shifts the analytical focus from isolated compounds to characteristic co-exposure profiles. However, because mixture-level epidemiological evidence currently derives from a single prospective cohort and no experiment has reproduced these mixtures at realistic combined doses, this review can assess the strength, limitations, and biological plausibility of the associations but cannot determine whether the mixtures themselves causally increase metabolic risk.

The mechanistic hypothesis connecting additive exposure to dysglycaemia centres on the intestinal epithelial barrier. Emulsifiers and certain thickeners disrupt the bilayered colonic mucus architecture [9], alter microbial community composition, and facilitate translocation of gram-negative bacterial wall components—principally lipopolysaccharide (LPS)—into systemic circulation. Chronic subclinical elevation of circulating LPS activates TLR4/CD14 signalling [10,11], driving NF-κB- and JNK1-dependent cytokine production that impairs insulin signalling through serine phosphorylation of insulin receptor substrate-1 (IRS-1) [12]. Non-nutritive sweeteners (NNSs) appear to act through partially distinct microbiome-dependent mechanisms [13,14]. Together, these pathways constitute a biologically plausible, though incompletely proven in humans, triad linking specific additive exposures to dysglycaemia and T2D risk. No human study has demonstrated this candidate chain end-to-end; its links derive from different experimental models and should not be read as an integrated causal pathway.

This review has four aims: (1) to evaluate the mixture-level epidemiological evidence linking food additive co-exposures to T2D; (2) to characterise the mechanistic sequence by which emulsifiers, NNS, and selected other additives may impair glucose homeostasis; (3) to assess the limitations of the current evidence base; and (4) to identify the design requirements for human intervention studies that could resolve outstanding mechanistic questions. This review is explicitly critical rather than confirmatory: evidence supported only by animal or in vitro data is identified as such, and genuinely conflicting findings are presented rather than minimised.

## 2. Materials and Methods

This article is a critical narrative review conducted in accordance with the Scale for the Assessment of Narrative Review Articles (SANRA) [15]. Its purpose was critical interpretive synthesis rather than exhaustive systematic identification or quantitative pooling. PubMed/MEDLINE, Embase, Web of Science Core Collection, Scopus, and CENTRAL were searched from database inception to 30 April 2026. A supplementary PubMed search on 26 July 2026 targeted newly indexed prospective cohorts and human interventions. Citation tracing and relevant EFSA, FDA, JECFA, and IDF documents supplemented the database searches. Search concepts combined “food additive*”, “additive mixture*”, named additive classes and compounds, the T2D concept (including “type 2 diabetes mellitus”), glycaemic outcomes, gut microbiota, intestinal barrier function, inflammation, and insulin signalling. Quotation marks denote phrase searches, whereas asterisks denote truncation to capture variant word endings; database-specific syntax was adapted where required. Prospective cohort and human intervention studies were prioritised, whereas animal, ex vivo, and in vitro studies were used to evaluate biological plausibility. English-language full texts, or records with sufficient English-language data for verification, were eligible when they addressed additive exposures and T2D, validated glycaemic outcomes, microbiota, barrier integrity, endotoxaemia, inflammatory signalling, incretin secretion, or insulin signalling. Selection and extraction were conducted narratively; no PRISMA record flow, duplicate independent screening, formal study-level risk-of-bias assessment, or GRADE rating was performed. Consequently, this article should not be interpreted as a systematic review. The final synthesis cites 106 sources, including approximately 55 original empirical reports together with reviews, methodological papers, and regulatory or public-health documents. Findings were organised by additive class and mechanistic pathway, and observational associations were explicitly distinguished from causal evidence. The interpretation follows SANRA principles of transparent searching, balanced synthesis, scientific reasoning, and explicit acknowledgement of limitations [15].

## 3. Food Additive Mixtures as Dietary Exposures

### 3.1. The Case for Moving Beyond Single-Additive Models

In real-world diets, food additives are not consumed in isolation. A single commercially processed food item may contain multiple distinct additives, and habitual consumers of UPFs can encounter many additive co-exposures across a day. The European Union currently approves over 330 food additives across 27 functional categories, including emulsifiers (E400–E495), preservatives (E200–E285), acidity regulators (E330–E380), sweeteners (E900–E967), and colourants [16]. Regulatory risk assessment has historically been conducted compound by compound against individual acceptable daily intake (ADI) thresholds; although a harmonised methodological framework for combined-exposure risk assessment of multiple chemicals now exists at EFSA [17], it has not yet been routinely operationalised for food additives, with no formal cumulative assessment groups established for most additive classes, leaving co-exposure evaluation largely absent from day-to-day regulatory practice.

The rationale for mixture-level analysis is not merely practical but scientific. Toxicology and mixture-risk assessment frameworks recognise that co-exposures may be additive, supra-additive (synergistic), or antagonistic, depending on whether co-exposed compounds share mechanistic targets, compete for the same transporters, or alter each other’s bioavailability [8,17]. When multiple additives act on overlapping biological substrates—such as the colonic mucus layer, tight junction proteins, or gut microbial communities—their combined effect may not be predictable from single-compound data. Adjacent work in the additive–oncology literature further illustrates the plausibility of co-exposure signals: NutriNet-Santé analyses of food additive emulsifiers and cancer risk [18] and of artificial sweeteners and cancer risk [19] provided the methodological groundwork for the mixture-level T2D analyses reviewed here. This consideration motivates a mixture-level empirical approach.

### 3.2. The NMF Mixture Paradigm: Evidence from the NutriNet-Santé Cohort

The first application of NMF to additive co-exposure data in a prospective T2D study was published by Payen de la Garanderie et al. in 2025 [8]. The cohort comprised 108,643 French adults (mean follow-up 7.7 years, SD 4.6; 79.2% women; mean age 42.5 years, SD 14.6), and 1131 incident T2D cases were detected during follow-up. Dietary exposure to 166 food additives was estimated from repeated 24-h dietary recalls linked to a comprehensive database of 3500 commercial food products with declared ingredient lists. NMF decomposed these additive intake data into five latent mixture profiles representing characteristic co-consumption patterns.

Mixture 2—dominated by emulsifiers (modified starches, pectin, guar gum, carrageenan, polyphosphates, and xanthan gum), the preservative potassium sorbate, and the colourant curcumin, found primarily in broths, dairy desserts, and sauces—was associated with an HR of 1.08 (95% CI: 1.02–1.15, *p* = 0.006) per one standard deviation increment in the NMF mixture score. The authors themselves described Mixture 2 as comprising “emulsifiers, preservatives, and a dye” [8]. Mixture 5—characterised by acidifiers (citric acid, sodium citrates, phosphoric acid, and malic acid), artificial sweeteners (acesulfame-K, aspartame, and sucralose), colourants (sulphite ammonia caramel, paprika extract, anthocyanins, and carnauba wax), and the emulsifiers arabic gum, guar gum, and pectin, found predominantly in artificially sweetened beverages and sodas—was associated with an HR of 1.13 (95% CI: 1.08–1.18, *p* < 0.001). No significant T2D association was observed for the three remaining mixture profiles [8].

Both associations remained statistically significant after adjustment for the Programme National Nutrition Santé Guidelines Score (PNNS-GS2), indicating that they were not explained by measured dietary quality in those models. However, statistical adjustment cannot establish independence from residual dietary-pattern or food-matrix confounding [8]. Exploratory interaction analyses identified both synergistic and antagonistic statistical interactions between individual additives, but these analyses do not establish biological synergy or mechanistic causality.

### 3.3. Why the Mixture Signal Demands Cautious Interpretation

A methodological distinction that bears sustained emphasis: NMF identifies additive clusters that statistically co-occur in habitual diets. It does not demonstrate that the additives within a cluster interact biologically, nor that any particular mixture member drives the observed association independently of others. Mixture scores are markers of a dietary pattern rather than validated biological synergy signals. The authors of the original study are explicit on this point [8].

Two specific interpretive challenges follow from this. First, the food matrices correlated with Mixture 2—broth, dairy desserts, fats and sauces—carry their own co-exposures in saturated fat, sodium, and refining-process contaminants that may partially drive the T2D signal independent of any additive effect. Second, Mixture 2 contains pectin and guar gum, both of which have independent human intervention evidence of glycaemic benefit when consumed as isolated viscous fibres (discussed in Section 8) [20,21]. Their presence in an epidemiologically harmful mixture most plausibly reflects co-occurrence in specific processed food matrices rather than independent glycaemic harm; this distinction is essential to avoid misrepresenting the mechanistic basis of the association. No published in vitro, animal, or human study has examined the combined biological effect of Mixture 2 or Mixture 5 components at simultaneously relevant concentrations. Experimental co-exposure designs remain the priority next step. The principal mixture-level findings are summarised in the first row of Table 1.

## 4. Epidemiological Evidence Linking Food Additives to T2D

### 4.1. Additive Mixtures and T2D Incidence

The Payen de la Garanderie 2025 NutriNet-Santé analysis [8], described in Section 3.2, was the first and, at the date of the final search, remained the only prospective investigation of additive mixtures as a single exposure unit for T2D incidence. Several design features support the robustness of the association: the large cohort (*n* = 108,643) provides adequate statistical power; incident T2D cases were detected through a validated algorithm incorporating self-reports, medical records, and glucose-lowering medication; adjustment was made for 25 covariates spanning demographic, anthropometric, dietary, and behavioural confounders; and the findings remained consistent across multiple sensitivity analyses including lag-time exclusions to address reverse causation.

The main limitations are inherent to the observational design. The NutriNet-Santé cohort is predominantly female (79%), educated, and French—characteristics that limit generalisability to diverse populations. Additive exposure was estimated from food product composition databases rather than biomarkers, introducing measurement error. Residual confounding by unmeasured dietary or lifestyle factors cannot be excluded. The NMF scores are continuous mixture-exposure indices derived from the same cohort, making the analysis exploratory with respect to mixture synergy. A more cautious interpretation is that Mixture 2 and Mixture 5 identify dietary patterns involving heavy co-use of specific additive categories, and that these dietary patterns—whether through the additives themselves, their food matrices, or both—carry elevated T2D risk in this population.

### 4.2. Emulsifiers and T2D Risk

The systematic epidemiological examination of dietary emulsifiers as individual exposures was initiated by Salame et al. in a 2024 NutriNet-Santé analysis (*n* = 104,139; 1056 incident T2D cases; mean follow-up 6.8 years) [22]. After adjustment for 25 covariates, higher intakes of total carrageenans, guar gum, tripotassium phosphate, sodium citrate, and xanthan gum were associated with incident T2D; the quantitative estimates are reported in Table 1 [22]. The associations were generally modest, and their heterogeneity across emulsifier subclasses argues against a uniform class effect. Estimates close to the null may be sensitive to modest residual bias, and adjusted absolute risk differences were not reported. Ex vivo evidence from Naimi et al. [28] indicates that lecithin—in contrast to carboxymethylcellulose (CMC), polysorbate 80 (P80), and carrageenan—does not substantially alter human microbiota composition or increase bioactive LPS and flagellin in MiniBioReactor Arrays, consistent with mechanistic heterogeneity within the emulsifier class. These compound-specific findings extend the same team’s earlier NutriNet-Santé analysis linking emulsifier exposure to incident cardiovascular disease [29], but there is no independent additive-specific multi-cohort replication of the NutriNet-Santé emulsifier–T2D signal.

The same limitations apply as for the broader NutriNet-Santé analyses: the cohort’s demographic specificity, dietary assessment based on repeated recalls, and inability to separate additive from food matrix effects. Persistence after adjustment for overall UPF intake indicates robustness to the measured covariates, not separation of the emulsifier signal from unmeasured variables or the food matrices that contain these compounds [30]. These findings support further mechanistic investigation, but do not establish causality.

### 4.3. Preservatives, Sweeteners, and Other Additives

The first large-scale prospective analysis of preservatives and T2D, by Hasenböhler et al. in 2026, examined 108,723 NutriNet-Santé participants and 1131 incident T2D cases [23]. Among 17 individually analysed preservatives, 12 were positively associated with incident T2D after correction for multiple testing [23]. The strongest associations involved potassium sorbate and sodium nitrite, while total, non-antioxidant, and antioxidant preservative exposures were also positively associated; quantitative estimates are reported in Table 1 [23]. These findings extend an earlier analysis of the same cohort in which additive-derived nitrites, particularly sodium nitrite, were associated with incident T2D, whereas additive-derived nitrates were not [25]. Positive associations for antioxidant additives, including sodium ascorbate and alpha-tocopherol, are counter-intuitive and may principally reflect food-matrix confounding because these compounds frequently co-occur with nitrites and saturated fat in processed meats. The authors characterise the data as hypothesis-generating [23]. Tertile comparisons describe the upper end of the exposure distribution and should not be translated directly into an average per-serving effect; continuous per-unit estimates were considerably smaller.

For NNS, the largest prospective NutriNet-Santé cohort analysis reported that higher total artificial sweetener intake and higher intakes of aspartame, acesulfame-K, and sucralose were each associated with incident T2D; compound-specific estimates are reported in Table 1 [24]. The comparatively smaller point estimate for sucralose should be noted. These associations may partly reflect reverse causation because individuals may switch to NNS in response to early weight gain or medical advice, even after lag-time sensitivity analyses. The wider NNS literature includes cardiometabolic signals beyond T2D, including a NutriNet-Santé cancer analysis [19] and prospective observations linking the sugar alcohol erythritol to major adverse cardiovascular events [31]; genotoxic effects of the sucralose metabolite sucralose-6-acetate have also been reported in vitro [32]. NNS epidemiology and mechanisms are examined in detail in Section 7.

A recently published NutriNet-Santé analysis of 108,723 participants and 1131 incident T2D cases reported positive associations for total food colouring additives and several individual colourants after false-discovery-rate correction, including caramel colours, beta-carotene, curcumin, and anthocyanins; quantitative estimates are provided in Table 1 [26]. As with the other NutriNet-Santé analyses, these findings are observational, non-independent of the shared cohort, and vulnerable to residual and food-matrix confounding. Evidence for acidifiers as independent T2D risk factors remains insufficient. Acidifiers appear in Mixture 5, but whether this reflects their own biological activity or co-occurrence with NNS in artificially sweetened beverages cannot be determined. The strongest mechanistic plausibility remains with selected emulsifiers through gut-barrier disruption and with specific NNS through microbiome-dependent glycaemic effects.

Taken together, the NutriNet-Santé analyses identify associations of incident T2D with two additive mixtures, selected emulsifiers, preservatives, food colouring additives, and non-nutritive sweeteners. Their directional coherence represents consistency across several exposure definitions applied to a substantially overlapping cohort, not replication across independent populations. All estimates remain observational and vulnerable to residual confounding, reverse causation where relevant, exposure misclassification, and food-matrix effects. The main findings are summarised in Table 1.

## 5. Mucus–Microbiota Interactions and Gut Barrier Dysfunction

### 5.1. The Colonic Mucus Bilayer as a Functional Barrier

The colonic epithelium is separated from the luminal microbiota by a bilayered mucus system assembled from the gel-forming MUC2 mucin secreted by goblet cells. The inner layer is densely packed, firmly adherent to the epithelial surface, and effectively devoid of viable bacteria under homeostatic conditions, whereas the outer layer—formed by proteolytic expansion of MUC2 networks—serves as the principal habitat for the luminal microbiota [9,33,34]. The intestinal microbial community itself is numerically comparable to the total number of human cells in the body [35,36], and its metabolic activity constitutes a distributed organ-like function that modulates host energy balance and glucose homeostasis [30,37,38]. The integrity of the inner layer determines whether luminal bacteria and their products can access the epithelial surface and trigger innate immune activation. This architecture is nutritionally dynamic: in a gnotobiotic mouse model transplanted with 14 human gut bacterial species, fibre deprivation drove mucus-foraging bacteria—including *Akkermansia muciniphila*, *Bacteroides thetaiotaomicron*, and *Bacteroides caccae*—to shift to mucosal glycans as alternative nutrient sources, thinning the inner mucus layer and enabling bacterial encroachment and pathogen penetration [39]. *A. muciniphila* is a keystone mucus-colonising organism with inverse associations with obesity and T2D risk in humans [40,41,42], and its depletion by dietary emulsifiers has been consistently observed in animal models. Soluble dietary fibre—abundant in whole foods but largely absent from UPF-heavy diets—sustains mucus layer integrity by providing fermentable substrate that diverts mucus-degrading bacteria away from host glycans [43,44]. Concurrent obesity-associated shifts in overall microbial community structure [45,46] compound this vulnerability.

### 5.2. Emulsifier-Mediated Mucus Erosion: Animal Evidence

The foundational mechanistic study linking dietary emulsifiers to gut barrier disruption was performed by Chassaing et al., who fed wild-type C57BL/6 mice CMC or P80 at approximately 1% in drinking water for 12 weeks [47]. Both emulsifiers produced: reduced microbial diversity; marked encroachment of the microbiota into the normally sterile inner mucus layer; elevated faecal flagellin and LPS; low-grade colonic inflammation; and a metabolic syndrome phenotype including fasting hyperglycaemia, adiposity, and features of insulin resistance. Germ-free mice fed the same emulsifiers showed no inflammatory or metabolic phenotype; faecal microbiota transplant (FMT) from emulsifier-treated conventional donors into germ-free recipients transferred both the inflammatory and metabolic phenotypes [47]. These FMT experiments establish microbiota-dependent causality for the metabolic effects in mice, though the dose employed (1% in drinking water) substantially exceeds estimated habitual human dietary exposures. *A. muciniphila* was substantially depleted in emulsifier-treated animals. Subsequent work by the same group linked chronic emulsifier exposure to colonic carcinogenesis in murine models, extending the low-grade inflammation phenotype to a pro-neoplastic dimension [48].

The mechanistic basis for mucus disruption involves the surfactant properties of amphiphilic emulsifiers: CMC and P80 reduce interfacial tension at the mucus–lumen boundary, lowering mucus viscosity and altering the penetrability threshold for bacterial access to the inner layer [30,47,49,50]. This mechanism is distinct from dietary fibre deprivation-driven degradation: emulsifiers actively destabilise mucin networks rather than passively depleting their substrate. An ex vivo analysis by Naimi et al. exposed a human microbiota maintained in MiniBioReactor Arrays to 20 dietary emulsifiers and confirmed that CMC, P80, carrageenan, and selected other emulsifiers produced lasting decreases in microbiota diversity and increases in bioactive LPS and flagellin [28]. Lecithin was among the few emulsifiers that did not significantly alter microbiota composition or pro-inflammatory potential in this system [28]. These data support mechanistic heterogeneity within the emulsifier class—a finding with direct implications for the interpretation of epidemiological emulsifier class analyses.

For carrageenan specifically, a converging body of work from Bhattacharyya, Tobacman, and colleagues has documented complementary cellular and animal findings: carrageenan impaired insulin signalling in HepG2 cells [51] and produced glucose intolerance and insulin resistance in C57BL/6J mice [51]. Regulatory review of carrageenan by the EFSA Panel on Food Additives concluded that additional data are needed to fully characterise safety in vulnerable subgroups [52], and a targeted narrative synthesis has summarised the parallel evidence base in inflammatory bowel disease and allergic contexts [53]. Broader historical experimental work in animals is also consistent with a pro-inflammatory gastrointestinal profile [54].

Important limitations apply to the animal data. Murine colonic mucus differs from human in composition, turnover rate, and microbiota-mucosal geometry. CMC doses in the Chassaing 2015 study exceed estimated human dietary exposure by a factor of 5–10 [47]. The metabolic syndrome phenotype in mice develops over 12 weeks of continuous exposure; whether equivalent exposure durations in free-living humans would be sufficient to produce measurable metabolic effects has not been established. No animal study has examined the combined effect of a realistic dietary emulsifier mixture at human-relevant doses.

### 5.3. Human Evidence: Emulsifier Feeding Trials

The only published human randomised controlled feeding trial of an individual emulsifier was conducted by Chassaing et al., who randomised 16 healthy adults in a parallel-arm, double-blind design (*n* = 9 emulsifier-free control; *n* = 7 CMC 15 g/day) for 11 days (NCT03440229) [55]. After 10 days of exposure (study day 14), microbiota composition differed between groups (PERMANOVA *p* = 0.002) and evenness was lower (*p* = 0.032; overall diet effect *p* = 0.070), whereas the Shannon-index difference did not reach statistical significance (*p* = 0.091; overall diet effect *p* = 0.151). Relative abundances of *F. prausnitzii* and *Ruminococcus* spp. decreased, whereas *Roseburia* spp. and Lachnospiraceae increased; faecal SCFAs and other microbiota-derived metabolites were depleted. Bacterial encroachment was observed in 2 of 7 CMC-treated participants (exploratory Fisher exact *p* = 0.175) [55]. Both arms lost approximately 1 kg during controlled feeding and showed comparable modest glycaemic improvements; no between-arm deterioration in glycaemic control or OGTT-derived insulin sensitivity was detected, although serum insulin declined modestly in the CMC arm. The trial supports short-term biological plausibility but is limited by *n* = 16, encroachment data from *n* = 7, an 11-day duration, and the absence of a sustained or patient-important glycaemic endpoint.

For carrageenan, the most rigorous human mechanistic evidence comes from a randomised double-blind crossover trial by Wagner et al. in 2024, which enrolled 20 males (mean age 27.4 ± 4.3 years; mean BMI 24.5 ± 2.5 kg/m^2^) and administered 250 mg carrageenan or placebo twice daily (morning and evening) for 2 weeks per condition, with a 4-week washout (NCT02629705) [56]. Primary analyses of overall insulin sensitivity showed no statistically significant differences between conditions across the full sample, and whole-sample CRP and IL-6 were also null. However, additional interaction analyses indicated that BMI moderated the treatment effect: in overweight participants, carrageenan exposure was associated with lower whole-body insulin sensitivity (OGTT-based insulin sensitivity index, *p* = 0.04 for interaction), reduced hepatic insulin sensitivity index (*p* = 0.04), elevated CRP and IL-6 (*p* = 0.02 for each BMI-by-treatment interaction), together with increased intestinal permeability (lactulose–mannitol *p* = 0.03; plasma zonulin *p* = 0.05) [56,57]. Activation of natural killer cells and elevated pro-inflammatory cytokine release were also observed in peripheral blood mononuclear cells after ex vivo carrageenan exposure. This small trial provides direct short-term human evidence consistent with carrageenan-related barrier and inflammatory changes, but the metabolic and inflammatory signals were interaction- or subgroup-dependent; its male-only sample and 2-week duration preclude population-level conclusions. An independent small open-label pilot in prediabetic adults (*n* = 13; 8 carrageenan-free vs. 5 usual-diet) reported within-arm improvements in HbA1c and HOMA-IR on the carrageenan-free diet relative to baseline, while between-arm differences reached statistical significance only for *C*-peptide and mechanistic markers (phospho-Ser-IRS1, phospho-AKT1, arylsulfatase B)—not for HbA1c or HOMA-IR themselves [58]. This hypothesis-generating pilot supports further testing in susceptible individuals but cannot establish a causal contribution of dietary carrageenan to dysglycaemia.

### 5.4. Tight-Junction Disruption and Epithelial Stress

The colonic epithelial tight junction complex—comprising claudins, occludin, zonula occludens proteins, and junctional adhesion molecules—regulates paracellular flux of ions and macromolecules including LPS fragments [59,60,61,62]. TNF-alpha and IFN-gamma, released during mucosal inflammatory responses to microbial encroachment, activate myosin light chain kinase (MLCK), reducing tight junction barrier function and creating a feed-forward loop between microbial contact, local inflammation, and permeability [59,63]. Butyrate, the principal SCFA product of colonic fibre fermentation, reinforces this barrier by enhancing tight junction protein expression (claudin-1, occludin, ZO-1) and inhibiting NF-κB activation in colonocytes [64]. SCFA depletion—as observed with CMC in the Chassaing 2022 human trial [55]—would be expected to reduce colonocyte energy supply and impair tight junction maintenance.

### 5.5. An Integrated but Unconfirmed Mechanistic Sequence

The following sequence is an integrative hypothesis assembled from separate experimental literatures rather than a pathway demonstrated end-to-end in a single human study. Food additive exposure, particularly to selected emulsifiers, may alter mucus–microbiota organisation through mucus erosion, microbial encroachment, and depletion of barrier-protective commensals. These changes may promote epithelial stress, local cytokine and MLCK activation, increased permeability, and translocation of microbial products such as LPS and flagellin. Systemic inflammatory signalling may then impair insulin action in liver, adipose tissue, and skeletal muscle. Each step has support in at least one experimental context, but most links derive from animal, cellular, or ex vivo models. The CMC trial lasted 11 days and showed no between-group glycaemic deterioration [55]; the carrageenan trial lasted 2 weeks and its adverse metabolic signals were subgroup- or interaction-dependent [56]. No human study has demonstrated the complete sequence from a defined additive exposure through endotoxaemia to clinically meaningful insulin resistance. The principal human trial findings relevant to this pathway are summarised in Table 2, while Table 3 provides a class-level synthesis of the broader mechanistic evidence.

## 6. Gut-Derived Inflammation and Insulin Resistance

### 6.1. Metabolic Endotoxaemia: Establishing the Mechanistic Bridge

The evidence in this section characterises the general link between endotoxaemia, innate immune signalling, and insulin resistance; it should not be interpreted as direct evidence that food additives activate the complete pathway in humans. Metabolic endotoxaemia refers to a chronic, subclinical elevation of circulating LPS—approximately two- to threefold above fasting baseline—without clinical sepsis. In Cani et al., 4 weeks of high-fat feeding raised plasma LPS into this range [71]. Continuous subcutaneous LPS infusion increased fasting glycaemia and insulinaemia and body, adipose-tissue, and liver weights to an extent similar to high-fat feeding; insulin resistance was detected in liver but not at whole-body level. Most LPS- and high-fat-diet-related metabolic effects were attenuated in CD14-mutant mice, supporting a central role for LPS/CD14 signalling in this murine model [71,72]. Selective enrichment of gut bifidobacteria [73] and prebiotic-driven improvement of GLP-2-mediated gut permeability [74] each attenuated elements of the high-fat-diet endotoxaemia–dysglycaemia phenotype, supporting a bidirectional microbiota–host relationship in mouse models.

Human data are consistent with the endotoxaemia hypothesis. In the FINRISK97 cohort (*n* = 7169; 462 incident T2D cases over 10 years), higher circulating endotoxin activity was prospectively associated with incident diabetes (hazard ratio 1.004 per unit LPS activity, 95% CI 1.001–1.007, *p* = 0.019), corresponding to a 52% higher risk of T2D in the highest versus lowest quartile of endotoxin activity (*p* = 0.013) after multivariable adjustment [75]. LPS-driven activation of the innate immune system in human adipose tissue has been demonstrated in obesity and T2D [10], providing a tissue-level correlate of the animal experiments. A single high-fat meal increased postprandial serum LPS by approximately 71% in healthy volunteers [76], implicating the intestinal barrier as a physiologically regulated checkpoint for LPS translocation. Complementary work by Amar et al. linked energy intake to circulating endotoxin in apparently healthy men and detected bacterial DNA in blood at the onset of human T2D [77,78]. These human data are observational or based on dietary challenge; specific causal demonstration of the chain from a defined food additive to elevated circulating LPS to insulin resistance remains absent from the human literature.

### 6.2. TLR4/CD14/MyD88 to IRS-1/IRS-2 Serine/Threonine Phosphorylation

The intracellular pathway linking LPS to insulin resistance is mechanistically well-characterised. LPS binds TLR4 in a co-receptor complex with CD14 and LPS-binding protein (LBP), initiating MyD88-dependent activation of IκB kinase-β (IKKβ) and c-Jun *N*-terminal kinase 1 (JNK1) [79]. IKKβ phosphorylates IκBα, liberating NF-κB to drive transcription of pro-inflammatory cytokines including TNF-alpha, IL-6, and IL-1β [80]. These cytokines can impair insulin signalling by promoting serine phosphorylation of IRS proteins. In one well-characterised cell-based pathway, TNF-alpha activates JNK1, which physically associates with IRS-1 and phosphorylates serine-307 (human equivalent: serine-312), reducing insulin receptor kinase-mediated tyrosine phosphorylation of IRS-1 and downstream PI3K/Akt activation [81,82]. Rui et al. demonstrated that both insulin and TNF-alpha stimulate IRS-1 Ser307 phosphorylation through distinct pathways, positioning IRS-1 serine phosphorylation as an important, though context-dependent, convergence node for inflammatory and nutrient-excess signals on insulin resistance [83]. In vivo evidence from Ser307Ala knock-in mice indicates that Ser307 phosphorylation can also act as a positive regulatory site rather than a purely inhibitory one—mutating this residue paradoxically exacerbates insulin resistance under high-fat feeding—so the pathway is best understood as one node within a wider IRS-1/IRS-2 serine/threonine phosphorylation network rather than as the single defining biochemical endpoint [67]. A broader synthesis of JNK signalling has more recently placed this kinase at the crossroads of obesity, insulin resistance, and cellular stress responses [12].

In JNK1-knockout obese mice, Hirosumi et al. demonstrated substantially improved insulin sensitivity and reduced adiposity, establishing JNK1 as a non-redundant driver of HFD-induced insulin resistance [84]. Shoelson et al. showed that constitutive IKKβ activation reproduced the insulin-resistant phenotype while salicylate-mediated IKKβ inhibition improved insulin sensitivity in obese humans, confirming translational relevance of the NF-κB arm of this pathway [80]. The same TLR4/CD14-mediated activation of the IKKβ/JNK1 inflammatory signalling network can disrupt IRS-1/IRS-2-dependent PI3K/Akt signalling across major insulin-sensitive tissues. In the liver, hepatic NF-κB activation promotes hepatic insulin resistance. In adipose tissue, macrophage M1 polarisation drives paracrine TNF-α and IL-6 signalling, thereby impairing adipocyte insulin responsiveness. In skeletal muscle, circulating inflammatory cytokines can interfere with GLUT4 translocation and reduce insulin-stimulated glucose uptake. Hotamisligil’s broader mechanistic synthesis established that this inflammatory signalling network is activated by the same molecular ligands—LPS, fatty acids, ceramides—regardless of origin, and that IRS-1 serine phosphorylation constitutes a common endpoint for obesity- and inflammation-driven insulin resistance [85]. Elevated TNF-alpha expression in adipose tissue from obese rodent models was among the first observations linking inflammation to insulin resistance [86], and low-grade systemic activation of innate immunity is now regarded as a core feature of T2D pathogenesis [11].

### 6.3. Microbial Metabolites as Potential Secondary Mediators

Beyond the LPS–endotoxaemia axis, gut dysbiosis may affect glucose metabolism through altered microbial metabolites. Faecal SCFAs and other microbiota-related metabolites were depleted after short-term CMC exposure in the human feeding trial [55]. Such depletion may reduce GPR41/43 signalling and could modify GLP-1 and PYY secretion and intestinal gluconeogenesis, but these downstream effects have not been demonstrated in human additive-exposure trials [64,87,88,89]. Microbiota-induced changes in bile-acid pools can alter FXR- and TGR5-mediated signalling, including regulation of hepatic bile-acid synthesis and enteroendocrine L-cell function [90], and bile-acid modifications have been reported after CMC and P80 exposure in experimental models and the short human CMC trial [47,55]. Tryptophan-derived indoles signal through the AhR in enterocytes and immune cells, influencing barrier integrity and inflammatory tone. Pharmacologically, metformin can remodel the gut microbiome, providing proof of concept that microbiota-targeted interventions may modulate systemic glucose homeostasis [91]. Thus, selected metabolite shifts have been measured in short human additive-exposure trials, but none has been shown to mediate a clinically meaningful glycaemic effect in humans; they remain candidate secondary mediators.

**Figure 1:**
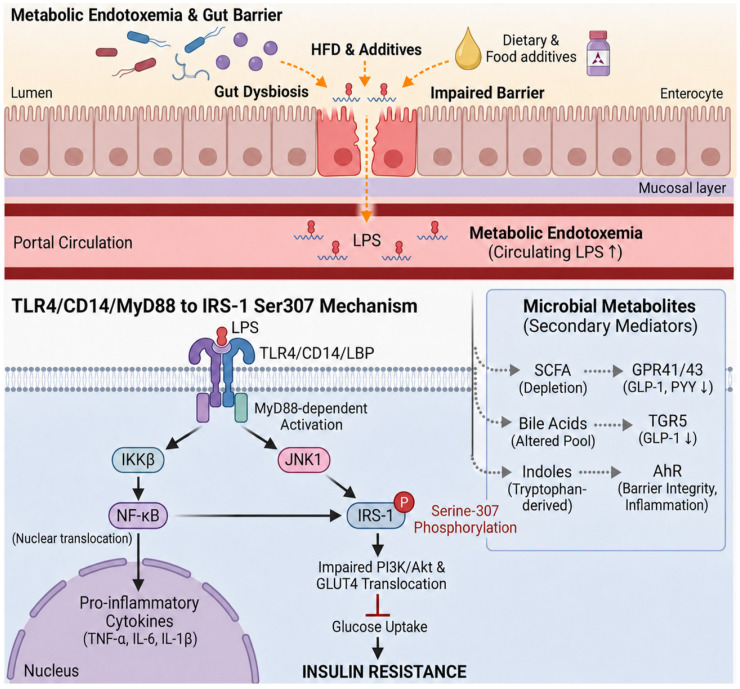
Evidence-tiered conceptual framework linking selected food additives to impaired glucose regulation. The upper part of the figure illustrates the proposed effects of high-fat dietary patterns and selected food additives on gut microbial composition, intestinal barrier integrity, and the translocation of lipopolysaccharide (LPS) into the circulation. Circulating LPS may interact with lipopolysaccharide-binding protein (LBP) and activate the TLR4/CD14/MyD88 signalling cascade, with downstream involvement of IKK, JNK, NF-κB, and pro-inflammatory cytokines. These inflammatory signals may interfere with insulin signalling through context-dependent changes in IRS-1 regulation, including Ser307 phosphorylation, and reduced activation of the PI3K/Akt pathway, thereby potentially contributing to insulin resistance and impaired glucose homeostasis. Alterations in microbial metabolites may represent additional secondary mediators connecting additive-related changes in the gut microbiota with systemic metabolic responses. Dashed orange arrows indicate additive-specific transitions that remain proposed or are supported predominantly by animal, in vitro, ex vivo, or limited short-term human evidence. Solid dark arrows represent more extensively characterised components of general LPS-triggered inflammatory and insulin-signalling pathways, whereas dotted grey arrows indicate candidate microbial-metabolite pathways for which the evidence remains incomplete or inconsistent. The figure integrates findings obtained from different experimental models and should not be interpreted as an uninterrupted causal pathway demonstrated in humans. In particular, no human study has yet confirmed the complete sequence from exposure to food additive mixtures through microbiome and intestinal-barrier alterations to clinically meaningful impairment of glucose metabolism. The image was generated using Nano Banana Pro (Gemini 3 Pro Image; Google DeepMind, Mountain View, CA, USA; accessed June 2026).

Figure 1 presents an evidence-tiered proposed framework linking additive- and diet-related barrier impairment to systemic inflammation and insulin resistance; it does not depict an experimentally verified end-to-end pathway in humans. Solid arrows denote better-characterised general LPS-triggered signalling, dashed arrows denote proposed additive-specific transitions, and dotted arrows denote candidate microbial-metabolite pathways. Table 3 provides the complementary class-level evidence summary.

## 7. Non-Nutritive Sweeteners and Individual Glycaemic Responses

### 7.1. NNS Are Not a Uniform Class

Non-nutritive sweeteners occupy a chemically and pharmacologically heterogeneous category. Saccharin (E954), aspartame (E951), acesulfame-K (E950), sucralose (E955), and steviol glycosides (E960) differ substantially in molecular structure, intestinal absorption, metabolic fate, and capacity to alter microbial communities [13,14]. Lumping these compounds as an undifferentiated class in mechanistic discussions obscures experimentally demonstrated heterogeneity that is directly relevant to risk assessment and public health communication. Specifically: saccharin and sucralose are not metabolically equivalent to aspartame or stevia, and the evidence reviewed below makes this distinction unavoidable.

### 7.2. Suez 2022: Human RCT with Translational Microbiome-Transfer Evidence

The most rigorous human mechanistic data linking NNS to glucose tolerance come from a 2022 Cell RCT by Suez et al. [65]. A total of 120 healthy, non-NNS-habituated adults were randomised to saccharin, sucralose, aspartame, stevia, glucose control, or no-supplement control for 2 weeks. Sweeteners were administered in commercial glucose-containing sachets at fixed doses below their respective ADIs [65]. Saccharin and sucralose worsened glycaemic responses, whereas aspartame and stevia produced no significant group-level change; quantitative results are provided in Table 2 [65]. Substantial inter-individual variability was observed, and identifiable subsets of responders occurred within every sweetener group [65]. FMT from NNS-consuming humans reproduced the glycaemic phenotypes in germ-free recipient mice in a sweetener-specific manner, supporting microbiome mediation within this translational model but not, by itself, establishing chronic causality in humans. Each NNS produced distinct stool and oral microbiome and plasma-metabolome changes, with sweetener-specific microbial signals particularly evident for sucralose.

These findings provide the strongest available short-term human evidence that saccharin and sucralose worsened glycaemic responses at below-ADI doses, while the paired FMT experiments support microbiome mediation in germ-free recipient mice. The foundational 2014 study established microbiota-dependent effects for saccharin in germ-free/FMT mouse experiments [68]; the 2022 trial added human randomisation and a translational FMT component, but it did not establish chronic microbiome-mediated causality in humans. Complementary animal work has shown that acesulfame-K alters gut microbial composition and body-weight trajectories in mice [69], while a short human trial found that sucralose acutely modified insulin and GLP-1 responses [70]. The 2022 RCT lasted 2 weeks and enrolled healthy, lean, non-NNS-habituated adults. Whether the observed responses persist, amplify, or attenuate with longer exposure in habitual users or in people with overweight, prediabetes, or established dysbiosis remains unknown.

### 7.3. Epidemiological Cohort Data and Compound Heterogeneity

The NutriNet-Santé NNS cohort analysis [24] provides the largest prospective additive-specific evidence. Higher total artificial sweetener intake and each of aspartame, acesulfame-K, and sucralose were associated with incident T2D; the compound-specific estimates are provided in Table 1 [24]. The smaller sucralose estimate and the discordance between the positive prospective aspartame association and the short-term null RCT finding remain important. Plausible explanations include exposure duration, metabolic risk, food-matrix and behavioural confounding, prior NNS habituation, residual reverse causation, or genuine effect modification by health state. Existing data cannot distinguish among these explanations.

The mechanistic responses to saccharin, sucralose, aspartame, acesulfame-K, and steviol glycosides are demonstrably non-uniform. Differences in intestinal absorption (sucralose poorly absorbed; aspartame hydrolysed to phenylalanine and aspartate; stevia glycosides converted to steviol by colonic microbiota), microbial contact profiles [69], and sweet-taste receptor specificity in enteroendocrine cells all contribute to compound-specific biological effects [88,89,92,93]. Genotoxic and gut-barrier-disrupting properties reported for the sucralose metabolite sucralose-6-acetate [32] and observational cardiovascular signals for the sugar alcohol erythritol [31] further underline that low-calorie sweeteners are not a mechanistically homogeneous category. Additional sources of inter-individual variability include baseline microbiome composition, background diet, metabolic status, and prior NNS habituation—which the Suez 2022 RCT identified as a significant effect modifier.

### 7.4. Negative and Discordant Human Evidence Across Additive Classes

Negative and discordant findings are central to interpretation. The CMC trial detected microbiome and metabolome changes without between-group deterioration in glycaemic control or OGTT-derived insulin sensitivity; the carrageenan crossover trial was null in the whole-sample primary insulin-sensitivity analysis; and the carrageenan-free pilot did not show between-group effects on HbA1c or HOMA-IR. Aspartame and stevia were neutral at group level in the Suez trial. In the 24-week SODAS trial, substituting water for artificially sweetened beverages in habitual consumers with T2D did not improve glycaemic outcomes [66]. Conversely, an independent Australian cohort found higher T2D incidence among daily consumers of artificially sweetened soft drinks [27]; this beverage-level exposure does not identify a responsible additive. Quantitative estimates are reported in Table 1 and Table 2. Together, these results argue against a uniform additive or class effect and separate biological plausibility from clinically meaningful metabolic harm.

### 7.5. Defensible Conclusions on NNS

The available evidence supports carefully bounded conclusions. In the 2-week Suez RCT, saccharin and sucralose worsened group-level glycaemic responses in healthy, non-NNS-habituated adults at below-ADI doses; paired human-to-mouse FMT experiments supported, but did not prove in humans, microbiome mediation. Whether this translates to clinically relevant chronic effects in real-world NNS users—particularly those at metabolic risk—remains unresolved. Aspartame and stevia were neutral at group level in that trial, whereas prospective cohort associations emerged over years of habitual exposure. Reverse causation and residual confounding remain plausible in the cohort data. NNS glycaemic responses should therefore be evaluated by compound, dose, duration, and host context rather than treating artificial sweeteners as a uniform class.

The available human intervention and mechanistic trials therefore provide important proof-of-concept evidence, but they remain short, small, and heterogeneous in population, exposure duration, and endpoints. The key human trials discussed above are summarised in Table 2. Accordingly, these trials support biological plausibility and selected short-term mechanistic effects, but they do not establish sustained, clinically meaningful deterioration in HbA1c, incident T2D, or other patient-important outcomes.

## 8. Context Dependence: Dose, Food Matrix, and Host Susceptibility

### 8.1. Not All Additives Are Metabolically Equivalent

The mixture paradigm should not be misread as evidence that all additives within an epidemiologically associated mixture are independently harmful. Direct experimental and mechanistic evidence is most developed—but incomplete—for selected emulsifiers and NNS, including CMC, P80, carrageenan, saccharin, and sucralose, and remains dominated by animal models and short human studies. Direct mechanistic evidence at dietary doses remains sparse for xanthan gum, modified starches, curcumin used as a colourant, and carnauba wax; the recent curcumin association [26] is observational. This ranking concerns biological plausibility, not proven chronic human metabolic harm. Several additives in Mixtures 2 and 5 may be markers of their food matrices rather than primary drivers. Broader dietary context matters: whole grains and their non-fibre bioactives are associated with protection against T2D through multiple, partially fibre-independent mechanisms [44], and dietary fibre intake more generally is associated with reduced systemic inflammation [94].

### 8.2. The Pectin and Guar Gum Paradox

An important interpretive challenge is the presence of pectin and guar gum in Mixture 2 (HR 1.08) [8]. Both have independent evidence of glycaemic benefit when consumed as isolated viscous fibres at pharmacologically relevant doses. A recent systematic scoping review of 134 human intervention studies of pectin (doses ranging from ~0.1 g/day up to ~50 g/day) reported that pectin consumption is consistently associated with reductions in post-prandial blood glucose and insulin peaks, increased satiety, and delayed gastric emptying, mechanistically consistent with high-viscosity slowing of carbohydrate absorption and, for fermentable fibres, GLP-1–mediated enhancement of satiety signalling [20]. For guar gum, a GRADE-assessed systematic review and meta-analysis of randomised clinical trials found significant reductions in HbA1c in adults with T2D following guar gum supplementation, although effects on fasting glucose and body weight were not consistent [21]. In the Naimi et al. ex vivo emulsifier panel, lecithin was the additive that showed neutral effects on human microbiota composition and pro-inflammatory potential, whereas various carrageenans and gums were reported to produce “particularly stark detrimental impacts” on microbiota composition and bioactive LPS/flagellin release [28]. The argument that Mixture 2’s pectin/guar-gum content is unlikely to be driving its T2D signal therefore rests on the independent human intervention evidence for isolated pectin and guar gum (see refs. [20,21]) rather than on Naimi’s MiniBioReactor Array data. Delzenne et al. identified gut microbiota as promising metabolic targets in T2D management, with prebiotic fibre supplementation featuring as a key intervention mechanism [95].

The most plausible explanation is that pectin and guar gum co-occur in Mixture 2 because they are used as industrial texturisers and stabilisers in the same food categories—commercial dairy-based desserts, reduced-fat sauces, processed broths—that also contain carrageenan, modified starches, and potassium sorbate. Their inclusion in the mixture reflects food matrix co-occurrence rather than independent glycaemic harm. The epidemiological association should explicitly not be interpreted as evidence against dietary pectin or guar gum as isolated fibre sources or as ingredients in whole or minimally processed foods. The industrial-matrix context in which these additives appear is distinct from their behaviour as isolated dietary fibres.

### 8.3. Dose and Regulatory Thresholds

Regulatory acceptable daily intake (ADI) values for food additives are conceptually defined as the amount that can be ingested daily over a lifetime without appreciable health risk; in practice, they are derived from no-observed-adverse-effect levels (NOAELs) in chronic (typically two-year) animal toxicological studies, divided by an uncertainty factor of 100 to account for interspecies and inter-individual variability [52,96]. The endpoints on which classical NOAEL determination has traditionally focused include body-weight change, clinical chemistry, gross and histopathological organ toxicity, carcinogenicity, and reproductive/developmental toxicity. The mechanistic evidence reviewed here raises a distinct question: whether chronic low-grade microbiome-mediated metabolic effects operate at below-ADI doses through pathways—gut microbiota composition, mucus–barrier integrity, incretin secretion, low-grade inflammation—that were not systematically evaluated in the toxicological studies used to derive current ADI values. The Suez 2022 NNS RCT administered fixed daily commercial-sachet doses that the authors defined as below the corresponding ADIs; the sachets also contained glucose, and glycaemic responses worsened in the saccharin and sucralose arms [65]. The Chassaing 2022 CMC trial used 15 g/day, within the range of realistic high-end dietary exposure [55]. The Wagner 2024 carrageenan trial used 250 mg twice daily, a dose achievable through consumption of multiple carrageenan-containing foods [56]. This convergence does not indicate that current ADI values are unsafe on their original endpoints; it identifies a potential paradigm gap—the classical ADI framework may not fully capture chronic microbiome-mediated metabolic effects that were not among the endpoints considered when the ADI values were derived.

### 8.4. Host Susceptibility and Food Matrix Effects

The Wagner 2024 carrageenan trial identified BMI as an effect modifier: insulin-sensitivity reductions were concentrated in the overweight subgroup [56]. This is consistent with a susceptibility model in which pre-existing metabolic vulnerability may amplify responses to additive-mediated gut-barrier perturbation. In the NutriNet-Santé emulsifier analysis, stronger T2D associations were observed in participants with higher overall UPF consumption; more broadly, obesity-related pathophysiology may modify metabolic susceptibility [22,97]. Suez 2022 reported associations between pre-exposure microbiome features and subsequent individual glycaemic responses, particularly for sucralose; these signatures are not yet clinically validated predictors [65].

In real food matrices, additives are embedded in complex environments—fats, proteins, water activity, competing polysaccharides—that alter their bioavailability and delivery to the intestinal epithelium. Carrageenan dispersed in a full-fat dairy product may behave differently from aqueous carrageenan in terms of mucus-layer interaction kinetics. CMC at 15 g/day in a fibre-rich dietary context would be expected to produce different microbiota effects than the same dose in a fibre-depleted UPF diet—a variable not systematically controlled in the Chassaing 2022 trial [55,98]. Background dietary fibre intake is a plausible important moderator of the emulsifier–gut-barrier hypothesis because fibre supports mucus integrity and SCFA-producing microbiota; however, this effect-modifying role has not been directly tested in human additive trials.

Overall, the mechanistic interpretation differs substantially across additive classes and depends on compound identity, food matrix, dose, and host susceptibility. A simplified class-by-class summary of the proposed mechanisms and evidence status is provided in Table 3.

## 9. Methodological Limitations

### 9.1. Causal Inference in Observational Studies

The most fundamental limitation of the current evidence base is the complete absence of a human randomised controlled trial with a food additive or additive mixture as the independent variable and incident T2D as the primary outcome. All epidemiological signals—from the NutriNet-Santé emulsifier, preservative, NNS, and mixture analyses—are prospective observational associations. Their persistence after extensive covariate adjustment is encouraging but does not establish causality. These reports are not independent cohort replications: the mixture, emulsifier, preservative, colourant, and NNS analyses used substantially overlapping NutriNet-Santé participant pools and closely related exposure-assessment infrastructure.

The specific problem of residual confounding is acute in additive research. Additive exposure is strongly correlated with overall UPF consumption, which is in turn correlated with higher energy intake, lower dietary fibre, lower socioeconomic status, and multiple behavioural factors [99]. Although the NutriNet-Santé analyses adjusted for overall UPF category and dietary quality scores, residual confounding by food-matrix co-exposures—saturated fat in emulsifier-containing products, nitrite in preservative-containing processed meats—cannot be excluded. The NMF approach partially addresses this by identifying mixture profiles independent of single-additive effects, but NMF scores are themselves dietary pattern markers: they do not constitute experimental manipulation. Characterising any NMF mixture association as evidence of additive causality rather than dietary pattern tracking requires the experimental designs described in Section 10. This concern is especially important for relative estimates close to the null, such as HRs of 1.03–1.15, because modest unmeasured or imprecisely measured confounding could materially alter them. Adjustment reduces measured confounding but does not make additive exposure independent of socioeconomic position, health behaviour, dietary pattern, or food matrix.

Most cohort reports provide relative estimates rather than harmonised adjusted absolute risk differences. In the mixture analysis, 1131 cases occurred among 108,643 participants, a crude cumulative proportion of approximately 1.04% over follow-up; however, HRs per standard-deviation increment cannot be converted into individual absolute risk without model-based baseline survival estimates. Individual clinical significance therefore remains uncertain, although a small effect could be relevant at population level if exposure is common.

### 9.2. Exposure Misclassification and Brand Reformulation

Additive intake estimation from dietary recall data linked to food-composition databases is subject to multiple sources of error. Ingredient-list databases rely on declared additives rather than measured concentrations, and product formulations can change during follow-up [8,100]. Biomarkers could improve exposure assessment but are not routinely available for most food additives. The direction of bias from dietary measurement error and time-varying reformulation is not guaranteed: non-differential error may attenuate associations, whereas correlated or differential error can bias estimates in either direction [101]. Consequently, the observed HRs should not be assumed to underestimate the true effects.

### 9.3. Separating Additives from Ultra-Processing

A conceptually important limitation is the difficulty of distinguishing additive-specific effects from those attributable to other attributes of UPFs—energy density, low fibre content, rapid digestibility, advanced glycation end-products, and disruption of food structure. Hall et al. conducted the only published controlled inpatient feeding trial of UPF versus unprocessed diets, demonstrating that ad libitum access to a UPF diet led to significantly higher energy intake and weight gain compared with an unprocessed diet matched for total energy, macronutrients, sugar, salt, and fat [102]. This study controlled macronutrients but not additives, making it impossible to attribute the effect to additives. Conversely, the NutriNet-Santé analyses show that additive mixture associations persist after adjustment for overall UPF NOVA category, but this adjustment cannot fully separate additive from food-structure effects within UPF. Whether additives are mechanistic contributors or simply markers of dietary patterns that plausibly cause T2D through other routes remains unresolved in the absence of experimental designs that independently manipulate additive content while holding other UPF attributes constant.

### 9.4. Human Mechanistic Trial Limitations

The three most informative human mechanistic trials—Chassaing 2022 (CMC, *n* = 16, 11 days) [55], Wagner 2024 (carrageenan, *n* = 20, 2 weeks) [56], and Suez 2022 (NNS, *n* = 120, 2 weeks) [65]—were designed around selected intermediate outcomes but remain too small, too short, and too demographically restricted to support subgroup or population-level metabolic conclusions. Effect sizes from small trials may be inflated by selection bias and regression to the mean; the carrageenan effect on insulin sensitivity was observed only in the overweight subgroup (*n* = 9–10) of an already small trial. No human mechanistic trial has examined endpoints in both sexes across BMI strata with long enough follow-up to observe progression of glycaemic deterioration. The endpoint heterogeneity across published trials—fasting glucose, HOMA-IR, OGTT-derived AUC, hyperinsulinaemic-euglycaemic clamp, HbA1c, continuous glucose monitoring—further limits cross-trial synthesis. The mechanistic literature is also concentrated in a small number of research groups, and independent laboratory replication remains limited.

### 9.5. Species Translation and Single-Additive Limitations

Murine gut physiology differs from human in colonic transit time, mucus layer thickness and glycosylation profile, and microbiome composition at the species level. CMC and P80 metabolic syndrome in wild-type mice [47] constitutes substantial mechanistic evidence but does not prove dose-equivalent effects in healthy-weight humans at dietary concentrations. More broadly, every mechanistic trial conducted to date has examined a single additive in isolation. The statistical co-occurrence patterns identified by NMF analysis predict that carrageenan, potassium sorbate, modified starches, and xanthan gum are consumed simultaneously; whether this co-exposure produces different gut barrier, microbiome, or metabolic effects than any single compound alone is entirely untested. This is the central experimental gap between the epidemiological mixture paradigm and its mechanistic validation.

### 9.6. Overall Certainty of Evidence

Epidemiological evidence: internally coherent but of limited causal certainty because additive-specific T2D signals derive predominantly from substantially overlapping NutriNet-Santé samples. Mechanistic evidence: moderate for biological plausibility of selected compounds in animal, cellular, and ex vivo models, but limited for an integrated pathway in humans. Human intervention evidence: limited for clinically meaningful metabolic effects because trials are small, short, and dominated by surrogate endpoints, with null, subgroup-dependent, and compound-specific findings. Overall interpretation: current evidence supports targeted hypothesis testing, not causal claims or additive-specific clinical recommendations. These descriptors are qualitative narrative judgments rather than formal GRADE ratings.

## 10. Priorities for Informative Human Studies

### 10.1. Study Design Requirements

The studies most needed are controlled feeding trials that directly manipulate additive content while holding other dietary variables constant. The key design requirements are: isoenergetic conditions; macronutrient-matched, fibre-matched, and food-structure-matched intervention and control arms; use of additive-containing versus additive-free formulations of the same food product rather than additive supplementation in additive-naive diets; realistic additive doses and combinations reflecting habitual co-exposures identified by NMF; adequate duration (minimum 8–12 weeks for metabolic outcomes; 6 months for HbA1c); and target populations enriched for metabolic susceptibility—overweight, prediabetes, or early T2D—rather than exclusively healthy volunteers. Stratification at randomisation by baseline BMI, insulin resistance status, and, if feasible, gut microbiome composition should be pre-specified as primary effect modifiers [55,56,65,102,103]. Factorial or dismantling designs should compare the full mixture, individual components, and mixture-minus-one conditions to distinguish joint effects from dominant-component effects.

### 10.2. Glycaemic Endpoints

Primary glycaemic endpoints should include: fasting plasma glucose and fasting insulin (enabling HOMA-IR calculation); 2-h OGTT glucose and insulin (with *C*-peptide to distinguish insulin secretion from clearance); and continuous glucose monitoring (CGM)-derived parameters including mean glucose, postprandial incremental AUC (iAUC), glucose variability indices, and time above range (TAR). For trials of 6 months or longer, HbA1c provides a validated measure of sustained glycaemic burden. The use of both fasting and dynamic glucose assessment addresses the possibility that additive effects operate preferentially on postprandial glucose handling—the mechanistic prediction from carrageenan-mediated GLP-1 inhibition [104]—rather than on fasting glucose alone.

### 10.3. Mechanistic Endpoints

Mechanistic endpoints should capture each step in the proposed sequence from gut barrier disruption to insulin resistance. Gut barrier integrity should be assessed by validated permeability tests (lactulose–mannitol test; plasma zonulin if validated methods are used consistently); endotoxaemia by LPS-binding protein (LBP) and soluble CD14 (sCD14), which are more stable than LPS itself; systemic inflammation by high-sensitivity CRP (hsCRP), IL-6, and TNF-alpha; insulin signalling by HOMA-IR and OGTT-derived indices; and enteroendocrine function by postprandial GLP-1, GIP, and glucagon. Gut microbiota should be characterised by shotgun metagenomics (preferred over 16S rRNA amplicon sequencing for functional inference) with reporting of diversity metrics, key species abundances, and relevant functional pathway representation including butyrate biosynthesis capacity. Selected microbial metabolites—faecal SCFA concentrations, relevant bile acid species, selected indoles—should be measured as secondary endpoints. Detailed dietary assessment throughout the trial should include fibre intake and overall UPF proportion, as these are primary effect moderators. Exposure and adherence should be validated by assay of intervention foods and, where available, compound-specific urinary or plasma metabolites. Microbiome and metabolomic samples should be collected at baseline, repeatedly during exposure, and after washout to establish temporal sequence and reversibility. A hyperinsulinaemic-euglycaemic clamp, ideally combined with stable-isotope tracers in a mechanistic subset, would provide gold-standard measurements of whole-body and hepatic insulin sensitivity.

### 10.4. Gap-Filling Research Priorities

Certain specific gaps are particularly informative to close. For emulsifiers: a larger (*n* ≥ 100), longer (12-week minimum) controlled feeding trial using a realistic mixture of CMC, carrageenan, and P80 at dietary concentrations in overweight/prediabetic adults, with the primary endpoint of HOMA-IR and secondary endpoints covering the full mechanistic sequence. For NNS: a 6-month RCT comparing saccharin and sucralose versus aspartame versus stevia versus sugar-free control in overweight individuals with prediabetes, with CGM as the primary endpoint and microbiome profiling to test for individual-response prediction. For preservatives: initial in vitro MiniBioReactor Array studies examining whether potassium sorbate, sodium nitrite, and calcium propionate—among the strongest epidemiological signals in Hasenböhler 2026 [23]—alter human microbiota composition and tight junction-associated gene expression at dietary concentrations. For mixtures: any experimental design comparing the biological effects of a NMF-informed combination of Mixture 2 additives versus each component alone—in vitro or in a controlled crossover—would constitute a first experimental test of the mixture synergy hypothesis.

Established anti-inflammatory or meal-timing interventions could serve as external comparators in separate metabolic studies, but additive-manipulation trials should avoid co-interventions that obscure the independent effect of additive reduction [105,106].

## 11. Conclusions

Taken together, epidemiological, human-intervention, and experimental evidence identifies food additive mixtures as a biologically plausible but unproven contributor to glucose dysregulation. Prospective signals for emulsifiers [22], preservatives [23], and additive mixtures [8], together with newer colourant evidence [26], arise from repeated analyses of substantially overlapping NutriNet-Santé samples rather than independent cohort replications. Preservative mechanisms remain especially uncertain [23]. Experimental support derives mainly from animal models and small, short-term human trials. Thus, neither mixture-level causality nor prevention of T2D through reducing specific additives has been established.

The central research priority is adequately powered, longer-term controlled feeding trials that manipulate realistic additive mixtures while matching overall diet and food matrix, and that integrate exposure validation with repeated microbiome, intestinal-barrier, inflammatory, and validated metabolic endpoints. Until such trials and independent additive-specific cohort replications are available, additive mixtures should be regarded as a testable risk hypothesis rather than an established cause of dysglycaemia.

## Figures and Tables

**Table 1:** Prospective cohort evidence linking food additive exposures to type 2 diabetes mellitus.

Additive Exposure	Population	Main Metabolic Outcome	Evidence Type and Interpretation	Main Limitation
Additive mixtures: Mixture 2 and Mixture 5	*n* = 108,643; 1131 incident T2D; NutriNet-Santé	Mixture 2 HR = 1.08 (1.02–1.15); Mixture 5 HR = 1.13 (1.08–1.18) per SD [8]	Prospective observational evidence; two real-world co-exposure patterns were associated with incident T2D after adjustment for measured diet quality and NOVA classification	Observational; single French cohort; mixture scores are dietary-pattern markers; residual confounding possible
Emulsifiers: carrageenans, guar gum, xanthan gum, tripotassium phosphate, sodium citrate	Prospective cohort; *n* = 104,139; 1056 incident T2D; NutriNet-Santé	Carrageenans HR = 1.03 (1.01–1.05) per 100 mg/day; guar gum HR = 1.11 (1.06–1.17), tripotassium phosphate HR = 1.15 (1.02–1.31), sodium citrate HR = 1.04 (1.01–1.07), and xanthan gum HR = 1.08 (1.02–1.14), each per 500 mg/day [22]	Prospective observational evidence; selected emulsifiers associated with T2D; supports compound-specific rather than uniform class effects	Observational; same cohort; additive estimation from dietary records/databases; food-matrix confounding
Preservatives: 12/17 individually examined	Prospective cohort; *n* = 108,723; 1131 incident T2D; NutriNet-Santé	Potassium sorbate HR = 2.15 (1.76–2.63); sodium nitrite HR = 1.50 (1.26–1.78); total preservatives HR = 1.47 (1.26–1.73); non-antioxidant preservatives HR = 1.49 (1.27–1.74); antioxidant preservatives HR = 1.40 (1.19–1.65), highest versus lowest tertile [23]	Prospective observational evidence; multiple preservatives associated with T2D; hypothesis-generating signal for a poorly characterised exposure class	Observational; strong food-matrix confounding; mechanisms largely unknown
Artificial sweeteners: aspartame, acesulfame-K, sucralose	Prospective cohort; *n* = 105,588; 972 incident T2D; NutriNet-Santé	Total NNS HR = 1.69; aspartame HR = 1.63; acesulfame-K HR = 1.70; sucralose HR = 1.34 [24]	Prospective observational evidence; all three analysed NNSs associated with incident T2D; sucralose estimate lower than aspartame/acesulfame-K	Observational; reverse causation and residual confounding likely despite sensitivity analyses
Nitrite/nitrate additives	Prospective cohort; *n* = 104,168; 969 incident T2D cases; NutriNet-Santé	Additive-derived nitrites HR = 1.53 (1.24–1.88); sodium nitrite HR = 1.54 (1.26–1.90), higher consumers versus non-consumers; additive-derived nitrates were not associated [25]	Prospective observational evidence; supports a nitrite-specific rather than nitrate-class association	Observational; overlapping NutriNet-Santé cohort; processed-meat matrix and residual confounding
Food colouring additives	Prospective cohort; *n* = 108,723; 1131 incident T2D cases; NutriNet-Santé	Total colours HR = 1.38 (1.17–1.63); total caramel HR = 1.43 (1.21–1.67); plain caramel HR = 1.46 (1.26–1.70); beta-carotene HR = 1.44 (1.23–1.68); curcumin HR = 1.49 (1.29–1.73); anthocyanins HR = 1.40 (1.17–1.68) [26]	Prospective observational evidence; multiple natural and synthetic colouring additives were associated with T2D	Observational; overlapping NutriNet-Santé cohort; no independent replication; food-matrix confounding
Artificially sweetened soft drinks (beverage-level proxy)	Melbourne Collaborative Cohort Study; *n* = 36,608 adults aged 40–69 years	At least once daily versus never or less than once monthly: IRR = 1.38 (95% CI 1.18–1.61) [27]	Independent prospective cohort evidence at beverage level; the association persisted after additional adjustment for BMI and waist-to-hip ratio	Does not identify a specific sweetener or additive; self-reported beverage exposure; residual confounding and reverse causation remain possible

Abbreviations: ASB, artificially sweetened beverage; CI, confidence interval; HR, hazard ratio; IRR, incidence rate ratio; NMF, nonnegative matrix factorisation; NNSs, non-nutritive sweeteners; NOVA, food-processing classification system based on the nature, extent, and purpose of processing; SD, standard deviation; T2D, type 2 diabetes mellitus. Note: All NutriNet-Santé rows use substantially overlapping participant samples and should not be interpreted as independent cohort replications.

**Table 2:** Human intervention and mechanistic trials of food additives relevant to glycaemic outcomes.

Additive Exposure	Design/Population	Key Finding	Main Caveat
Carboxymethylcellulose (CMC) [55]	Controlled-feeding RCT; *n* = 16 healthy adults; 11 days; 15 g/day CMC	After 10 days of exposure (study day 14), microbiota composition differed between groups (PERMANOVA *p* = 0.002); evenness was lower (*p* = 0.032; overall diet effect *p* = 0.070), whereas the Shannon-index difference was not significant (*p* = 0.091; overall diet effect *p* = 0.151). *F. prausnitzii* decreased and bacterial encroachment occurred in 2/7 CMC-treated participants (exploratory *p* = 0.175). No between-group deterioration in glycaemic control or OGTT-derived insulin sensitivity was detected.	Very small and short; no direct permeability measure or sustained, patient-important glycaemic endpoint
Carrageenan [56]	Double-blind crossover RCT; *n* = 20 males; 250 mg twice daily for 2 weeks	Overall insulin sensitivity and whole-sample CRP and IL-6 were null. BMI-by-treatment interactions occurred for OGTT-derived insulin sensitivity (*p* = 0.04), HOMA2-IR (*p* = 0.01), hepatic insulin sensitivity (*p* = 0.04), CRP (*p* = 0.02), and IL-6 (*p* = 0.02); permeability increased overall (lactulose–mannitol *p* = 0.03; zonulin *p* = 0.05).	Small male-only trial; subgroup-driven; short duration
Non-nutritive sweeteners: saccharin, sucralose, aspartame, stevia [65]	Parallel-arm RCT; *n* = 120 healthy non-NNS users; 2 weeks; below-ADI doses	At week 2, GTT iAUC increased from baseline with saccharin by 811.2 mg·dL^−1^·min (95% CI 190.6–1432; *p* = 0.0094) and with sucralose by 976.3 mg·dL^−1^·min (95% CI 231.3–1721; *p* = 0.0092); aspartame and stevia produced no significant group-level change.	Healthy short-term trial; effects may differ in habitual or at-risk users
Carrageenan-free diet [58]	Open-label pilot RCT; *n* = 13 prediabetic adults; 12 weeks	Within the carrageenan-free arm, HbA1c decreased (*p* = 0.006) and HOMA-IR decreased (*p* = 0.026). Between-group changes were significant for *C*-peptide (*p* = 0.007), phospho-Ser-IRS1 (*p* = 0.038), phospho-AKT1 (*p* = 0.0012), and arylsulfatase B (*p* = 0.0008), but not for HbA1c or HOMA-IR.	Very small, open-label, no biomarker verification
Artificially sweetened beverages [66]	Parallel-arm RCT; *n* = 181 adults with type 2 diabetes mellitus and habitual ASB use; 24 weeks; 24 oz/day water substitution versus maintained ASB	Water substitution did not improve glycaemic measures. Mean 24-week HbA1c change was 0.29 percentage points higher in the water arm than in the ASB arm (SE 0.12; *p* = 0.013); secondary glycaemic outcomes were null.	Beverage-substitution trial in habitual users, not an isolated or compound-specific additive trial

Abbreviations: ADI, acceptable daily intake; ASB, artificially sweetened beverage; BMI, body mass index; CI, confidence interval; CMC, carboxymethylcellulose; CRP, *C*-reactive protein; FMT, faecal microbiota transplantation; *F. prausnitzii*, *Faecalibacterium prausnitzii*; GTT, glucose tolerance test; HbA1c, glycated haemoglobin; HOMA-IR, homeostatic model assessment of insulin resistance; HOMA2-IR, updated homeostatic model assessment of insulin resistance; iAUC, incremental area under the curve; IL-6, interleukin-6; NNS, non-nutritive sweetener; OGTT, oral glucose tolerance test; PERMANOVA, permutational multivariate analysis of variance; RCT, randomised controlled trial; SCFAs, short-chain fatty acids; SE, standard error.

**Table 3:** Simplified mechanistic summary by additive class.

Additive Class	Examples	Main plausible Mechanism	Evidence Status	References
Emulsifiers/thickeners	CMC, polysorbate 80, carrageenan	Mucus disruption, microbiota encroachment, gut-barrier impairment, low-grade inflammation, IRS-1/IRS-2 signalling disruption	Substantial animal evidence; limited short-term human mechanistic evidence	[28,47,51,55,56,67]
Non-nutritive sweeteners	Saccharin, sucralose, aspartame, acesulfame-K, steviol glycosides	Compound-specific microbiome changes affecting postprandial glucose responses	Moderate short-term mechanistic evidence; clinically limited and compound-specific	[13,14,24,65,68,69,70]
Preservatives	Potassium sorbate, sodium nitrite, propionates, sodium ascorbate	Unclear; possible microbiome effects, nitrosative stress, and food-matrix confounding	Emerging; mainly epidemiological	[23,25]
Gums/viscous fibre-like additives	Pectin, guar gum, xanthan gum, arabic gum	Context-dependent: isolated fibre benefits, but co-occurrence in UPF mixtures may mark harmful food matrices	Mixed; context-dependent	[8,20,21,28]
Other mixture components	Acidifiers, colouring additives, modified starches	Direct mechanisms remain uncertain; food matrix, beverage vehicle, pH, or starch digestibility may contribute	Emerging epidemiological evidence for colouring additives; weak direct mechanistic evidence	[8,16,22,26]

Abbreviations: CMC, carboxymethylcellulose; GLP-1, glucagon-like peptide-1; IRS, insulin receptor substrate; NNSs, non-nutritive sweeteners; P80, polysorbate 80; T2D, type 2 diabetes mellitus; UPF, ultra-processed food.

## Data Availability

No new data were created or analysed in this study. Data sharing is not applicable to this article.

## Abbreviations

The following abbreviations are used in this manuscript:

ADI	Acceptable daily intake
AhR	Aryl hydrocarbon receptor
CD14	Cluster of differentiation 14
CGM	Continuous glucose monitoring
CMC	Carboxymethylcellulose
FMT	Faecal microbiota transplantation
FXR	Farnesoid X receptor
GIP	Glucose-dependent insulinotropic polypeptide
GLP-1/GLP-2	Glucagon-like peptide-1/glucagon-like peptide-2
GLUT4	Glucose transporter type 4
GPR41/43	G protein-coupled receptors 41 and 43
HbA1c	Glycated haemoglobin A1c
HFD	High-fat diet
HOMA-IR	Homeostatic model assessment of insulin resistance
hsCRP	High-sensitivity *C*-reactive protein
iAUC	Incremental area under the curve
IκBα	Nuclear factor kappa-B inhibitor alpha
IKKβ	IκB kinase beta
IL-1β/IL-6	Interleukin-1 beta/interleukin-6
IRS-1/IRS-2	Insulin receptor substrates 1 and 2
JNK1	c-Jun *N*-terminal kinase 1
LBP	Lipopolysaccharide-binding protein
LPS	Lipopolysaccharide
MLCK	Myosin light-chain kinase
MUC2	Mucin 2
MyD88	Myeloid differentiation primary response 88
NF-κB	Nuclear factor kappa-light-chain-enhancer of activated B cells
NMF	Nonnegative matrix factorisation
NNS	Non-nutritive sweetener(s)
NOAEL	No-observed-adverse-effect level
NOVA	Food-processing classification system based on the nature, extent, and purpose of processing
OGTT	Oral glucose tolerance test
P80	Polysorbate 80
PI3K/Akt	Phosphoinositide 3-kinase/protein kinase B pathway
PNNS-GS2	Programme National Nutrition Santé Guidelines Score 2
PYY	Peptide YY
sCD14	Soluble cluster of differentiation 14
SCFA	Short-chain fatty acid(s)
T2D	Type 2 diabetes mellitus
TAR	Time above range
TGR5	Takeda G protein-coupled receptor 5
TLR4/TLR5	Toll-like receptors 4 and 5
TNF-α	Tumour necrosis factor-alpha
UPF	Ultra-processed food
ZO-1	Zonula occludens-1
